# Supplementary material for: Auranofin Targeting the NDM-1 Beta-Lactamase: Computational Insights into the Electronic Configuration and Quasi-Tetrahedral Coordination of Gold Ions
Source: Pharmaceutics. 2023 Mar 18;15(3):985. doi: 10.3390/pharmaceutics15030985 (PMC10057648; doi:10.3390/pharmaceutics15030985)
Supplement: Supplementary file 1 [file pharmaceutics-15-00985-s001.zip › pharmaceutics-2280198-supplementary.pdf]

Supporting Information

Auranofin targeting the NDM-1 beta-lactamase: Computational insights into the electronic configuration and quasi-tetrahedral coordination of gold ions

Iogann Tolbatov,* Alessandro Marrone

[a] Institute of Chemical Research of Catalonia (ICIQ), The Barcelona Institute of Science and Technology, Tarragona, 43007, Spain

[b] Dipartimento di Farmacia, Università degli Studi “G. D'Annunzio” Chieti-Pescara, Via dei Vestini, 66100 Chieti, Italy

*Corresponding author: tolbatov.i@gmail.com

Table S1. Experimentally measured Au-Au distances in crystal structures.

Crystal structures	Au-Au distance, A
$[Au_2(hpp)_2Cl_2]$, hpp = 1,3,4,6,7,8-hexahydro-pyrimido[1,2-a]pyrimidinate	2.47 [1]
$[Au_2(2,6\text{-}Me_2\text{-form})_2Cl_2]$, form=formamidinate	2.52 [2]
$[Au_2(2,6\text{-}Me_2\text{-form})_2]$, form=formamidinate	2.71 [2]
$[Au(SCN)_2]$	3.00-3.26 [3], 3.17 [4]
bacterial urease from *Sporosarcina pasteurii* (SPU) crystallized in presence of $Au(PEt_3)_2Cl$	3.18 [5]
bacterial urease from *Sporosarcina pasteurii* (SPU) crystallized in presence of $Au(PEt_3)I$	3.22 [5]
Dinuclear gold triphenylphosphine	3.29 [6]
(4-isocyano-4′-propoxy-1,1′-biphenyl)gold(I) chloride	3.42 [7]
chloro-(1-decyl-3-methyl-2,3-dihydro-1H-benzimidazol-2-ylidene)-gold(I)	3.49 [8]

Table S2. The Mulliken atomic spin densities on each gold metal center with the respective coordinating protein atoms, and on the hydroxide or water oxygen are reported for the systems with multiplicity > 1, i.e. **B**, **D**-**F**, and **H**. All values in a.u.

Atom	**B**	**D**	**E**	**F**	**H**
Au1	0.47	1.02	1.32	0.52	0.53
N(His122)	0.00	0.14	0.13	0.12	0.12
N(His189)	0.00	0.13	0.14	0.12	0.12
N(His120)	0.00	0.00	0.01	0.06	0.07
Au2	0.94	0.16	1.12	0.00	0.39
S(Cys208)	0.34	0.17	0.67	0.00	0.42
N(His250)	0.02	0.00	0.18	0.00	0.14

O(Asp124)	0.02	0.05	0.01	0.00	0.05
O(OH)	0.18	0.30	0.36	0.17	0.17
O(water)	0.03	0.02	0.03	0.00	0.00

References

[1] Irwin, M.D., Abdou, H.E., Mohamed, A.A., Fackler Jr, J.P., 2003. Synthesis and X-ray structures of silver and gold guanidinate-like complexes. A Au (ii) complex with a 2.47 Å Au–Au distance. Chemical Communications, (23), 2882-2883.

[2] Abdou, H.E., Mohamed, A.A., Fackler, J.P., 2005. Synthesis and X-ray structures of dinuclear and trinuclear gold (I) and dinuclear gold (II) amidinate complexes. Inorganic Chemistry, 44(2), 166-168.

[3] Coker, N.L., Krause Bauer, J.A., Elder, R.C., 2004. Emission energy correlates with inverse of gold–gold distance for various $[Au(SCN)_2]^-$ salts. Journal of the American Chemical Society, 126(1), 12-13.

[4] Kumar, K., Stefanczyk, O., Chorazy, S., Nakabayashi, K., Ohkoshi, S.I., 2022. Ratiometric and colorimetric optical thermometers using emissive dimeric and trimeric $\{[Au(SCN)_2]^-\}_n$ moieties generated in d–f heterometallic assemblies. Angewandte Chemie, 134(20), e202201265.

[5] Mazzei, L., Massai, L., Cianci, M., Messori, L., Ciurli, S., 2021. Medicinal Au (i) compounds targeting urease as prospective antimicrobial agents: unveiling the structural basis for enzyme inhibition. Dalton Transactions, 50(40), 14444-14452.

[6] Johnson, A., Marzo, I., Gimeno, M.C., 2020. Heterobimetallic propargyl gold complexes with π-bound copper or silver with enhanced anticancer activity. Dalton Transactions, 49(33), 11736-11742.

[7] Yamada, S., Rokusha, Y., Kawano, R., Fujisawa, K., Tsutsumi, O., 2017. Mesogenic gold complexes showing aggregation-induced enhancement of phosphorescence in both crystalline and liquid-crystalline phases. Faraday Discussions, 196, 269-283.

[8] Sathyanarayana, A., Siddhant, K., Yamane, M., Hisano, K., Prabusankar, G., Tsutsumi, O., 2022. Tuning the Au–Au interactions by varying the degree of polymerisation in linear polymeric Au (i) N-heterocyclic carbene complexes. Journal of Materials Chemistry C, 10(15), 6050-6060.
